# Targeting ferroptosis: a novel insight into thyroid cancer therapy

**DOI:** 10.3389/fendo.2025.1527693

**Published:** 2025-06-03

**Authors:** Xinyao Liu, Liangkai Wang, Xuehua Xi, Tongtong Zhou, Zhe Sun, Bo Zhang

**Affiliations:** ^1^ Department of Ultrasound, China-Japan Friendship Hospital, Beijing, China; ^2^ National Center for Respiratory Medicine; State Key Laboratory of Respiratory Health and Multimorbidity; National Clinical Research Center for Respiratory Diseases; Institute of Respiratory Medicine, Chinese Academy of Medical Sciences; Department of Ultrasound, Center of Respiratory Medicine, China-Japan Friendship Hospital, Beijing, China

**Keywords:** ferroptosis, iron, lipid peroxidation, thyroid cancer, targeted therapy

## Abstract

There is a continuous increase in the incidence of thyroid cancer. A deeper understanding of the molecular mechanisms of thyroid cancer could significantly improve thyroid cancer management. Newly discovered type of programmed cell death, ferroptosis, has been demonstrated to play a crucial role in many cancers. Mounting evidence shows that there is a close association between ferroptosis and thyroid cancer, which offer a promising therapeutic strategy for thyroid cancer. Ferroptosis is expected to emerge as a novel therapeutic target. Regrettably, the exact role of ferroptosis in thyroid cancer is not yet completely understood. Further, there is currently no summary of ferroptosis in thyroid cancer progression and treatment. Hence, in this review, we aim to revisit the pathological process of thyroid cancer and reveal the role of ferroptosis in thyroid cancer. In addition, we provide evidence that ferroptosis inducers could suppress the growth of thyroid cancer cells. Lastly, we discuss the potential application of ferroptosis inducers in thyroid cancer treatment, as well as possible impediments and corresponding strategies. The relationship between ferroptosis and thyroid cancer will be better understood through this review, which may offer a novel insight into thyroid cancer therapy.

## 1 Introduction

About 94.5% of endocrine tumors are thyroid cancers (1). According to the GLOBOCAN 2020 database by the WHO International Agency for Research on Cancer, the incidence of thyroid cancer is the ninth highest in the world (2, 3). The incidence of thyroid cancer has increased by about 4% per year in recent years, making it one of the few cancers with a rising incidence (4–6). Although certain therapeutic methods, including surgery, radiotherapy and chemotherapy, have been developed for thyroid cancer management, the recurrence rate remains high and the prognosis of some patients is poor (7, 8). Therefore, exploring new therapeutic targets and drugs is an important research direction in thyroid cancer management.

Ferroptosis is a newly identified type of programmed cell death and was proposed by Dixon et al. to describe the cell death triggered by RAS-selective lethal compounds due to its unique morphological, biochemical, and genetic features (9). Ferroptosis inducers can cause selective lethality in tumor cells that harbor RAS mutations (9). Approximately 40–50% of follicular thyroid cancers (FTC), 20–40% of poorly differentiated thyroid cancers and anaplastic thyroid cancers (ATC), and 10–20% of papillary thyroid cancers (PTC) have RAS mutations (10). Previous research has demonstrated that inducing ferroptosis can inhibit the proliferation of thyroid cancer cells (11). There is currently no comprehensive summary of the role of ferroptosis in thyroid cancer. Thus, in this review, the roles of key ferroptosis regulators, including iron, glutathione peroxidase 4 (GPX4), glutathione (GSH), and dipeptidyl-peptidase-4 (DPP4), in thyroid cancer are discussed. Furthermore, we summarize the agents related to ferroptosis that are used for thyroid cancer treatment. Lastly, the potential application of ferroptosis inducers in thyroid cancer treatment is proposed, and possible impediments and corresponding strategies are listed.

## 2 Ferroptosis and its core mechanisms

In 2012, Dixon et al. named ferroptosis as a new type of cell death (9). Ferroptosis differs from other types of programmed cell death, characterized by lipid peroxidation and iron overload (12). The morphological features of ferroptosis include shrunken mitochondria, reduced or diminished mitochondrial cristae, condensed mitochondrial membrane densities, and ruptured mitochondrial outer membranes (12, 13). The biochemical characteristics of ferroptosis involve iron overload, reactive oxygen species (ROS), and lipid peroxidation, which lead to damage and disorganization of the cell membrane by producing numerous alkyl oxygen radicals (14, 15).

The regulatory mechanism of ferroptosis is very complex, involving multiple metabolic pathways. The initiation and execution of ferroptosis are mainly related to the metabolism of amino acids, lipids, and iron.

### 2.1 Amino acid metabolism

**Figure 1:**
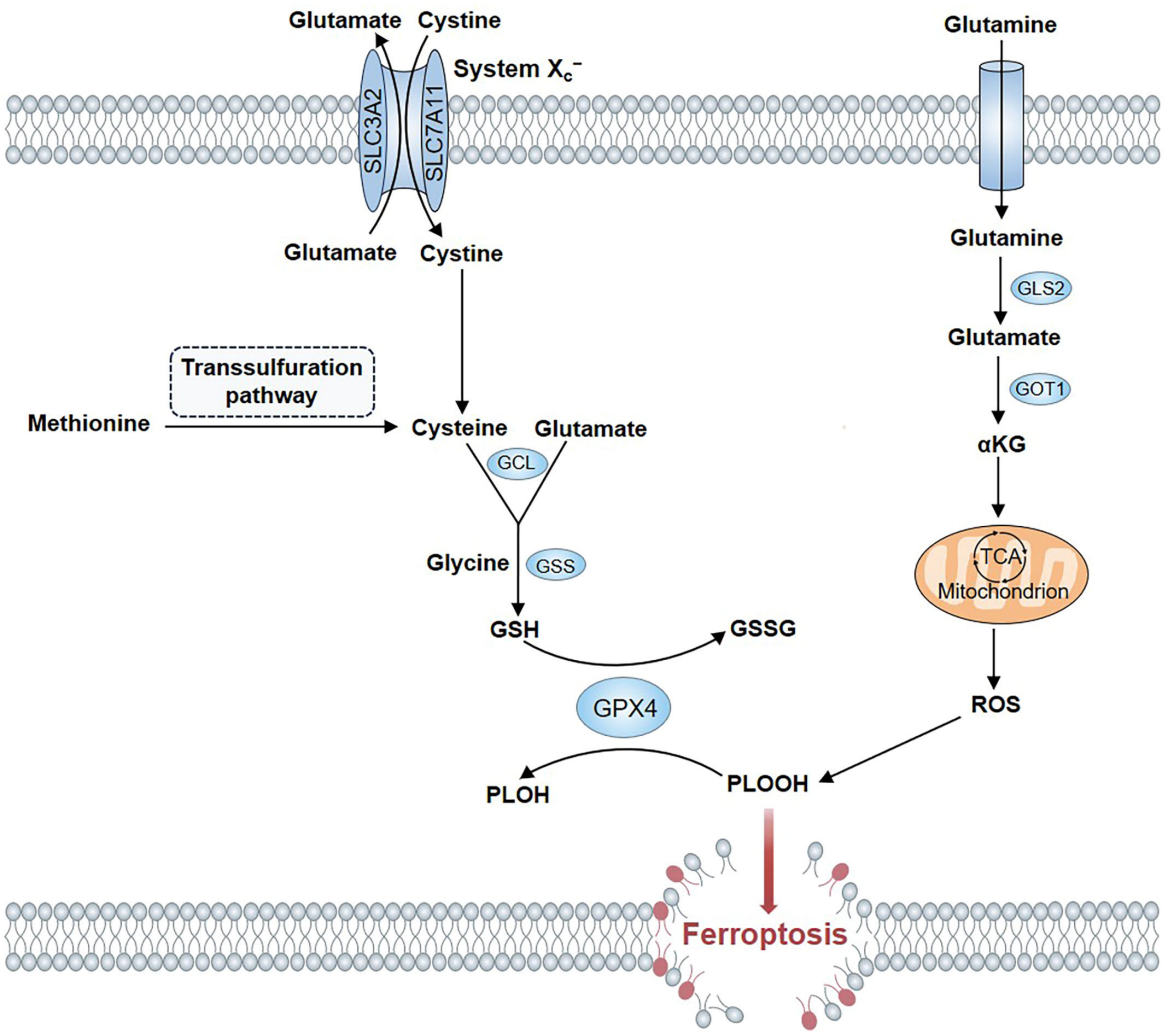
Amino acid metabolism in ferroptosis. The cystine/glutamate transporter allows the uptake of cystine via exchange with intracellular glutamate across the cell membrane in a 1: 1 ratio. Cystine enters into the cell and is converted to cysteine. Certain cells synthesize cysteine from methionine via the transsulfuration pathway. Cysteine is used to synthesize GSH in two steps under the catalysis of cytosolic enzymes GCL and GSS. GPX4 converts two molecules of GSH to GSSG and simultaneously reduce PLOOHs to nontoxic PLOHs in the membrane. α-ketoglutarate, a product of glutaminolysis, is a metabolic intermediate for promoting ferroptosis by producting ROS or lipid.

The regulation of ferroptosis is closely associated with amino acid metabolism (**Figure 1**). The cystine/glutamate antiporter system X_c_^−^ allows cystine to be taken up via 1:1 exchange with intracellular glutamate (16). Cystine enters into the cell and is converted to cysteine (15). Glutamate-cysteine ligase and glutathione synthetase (GSS) catalyze the synthesis of GSH from cysteine, glutamate, and glycine in two steps (17). As an antioxidant, GSH can specifically scavenge ROS. Cysteine, the least abundant of the three amino acids essential for GSH synthesis, serves as the rate-limiting factor in this biosynthetic process (18). As the availability of cysteine restricts GSH synthesis, certain cells synthesize cysteine from methionine via the transsulfuration pathway, thereby bypassing the need for cystine import through the system X_c_^−^ (17). Therefore, system X_c_^−^ inhibitors cannot induce ferroptosis in these cells. The biological activity of GPX4 is dependent on GSH. Two molecules of GSH are converted into oxidized GSH (GSSG) by GPX4 and phospholipid hydroperoxides are reduced to nontoxic phospholipid alcohols by GPX4 simultaneously, which prevents the accumulation of toxic lipid peroxides (19, 20). Therefore, inhibiting GSH and GPX4 activity results in the accumulation of lipid peroxidation and, eventually, ferroptosis (21).

Ferroptosis is also regulated by glutamine and glutamate (22). System X_c_^−^ activity can be inhibited by high extracellular concentrations of glutamate, consequently inducing ferroptosis. This may explain why a high concentration of glutamate in the nervous system may lead to toxic effects (9, 23). Glutaminolysis is involved in ferroptosis. However, not all pathways of glutaminolysis lead to ferroptosis. The initial step in glutaminolysis process involves the conversion of glutamine into glutamate. Glutaminase 1 (GLS1) and GLS2 catalyze glutamate production. Despite the structural and enzymatic similarity between GLS1 and GLS2, only GLS2 contributes to ferroptosis (22). Glutamate is converted to α-ketoglutarate (αKG) by glutamic oxaloacetic transaminase 1-mediated transamination. Finally, αKG induces ferroptosis by generating lipids or ROS (22, 24). Therefore, glutaminolysis-targeted therapy may provide a new idea to treat organ injury mediated by ferroptosis. In fact, research have suggested that inhibiting glutaminolysis could improve brain hemorrhage and kidney injury in experimental models (25, 26).

### 2.2 Lipid metabolism

**Figure 2:**
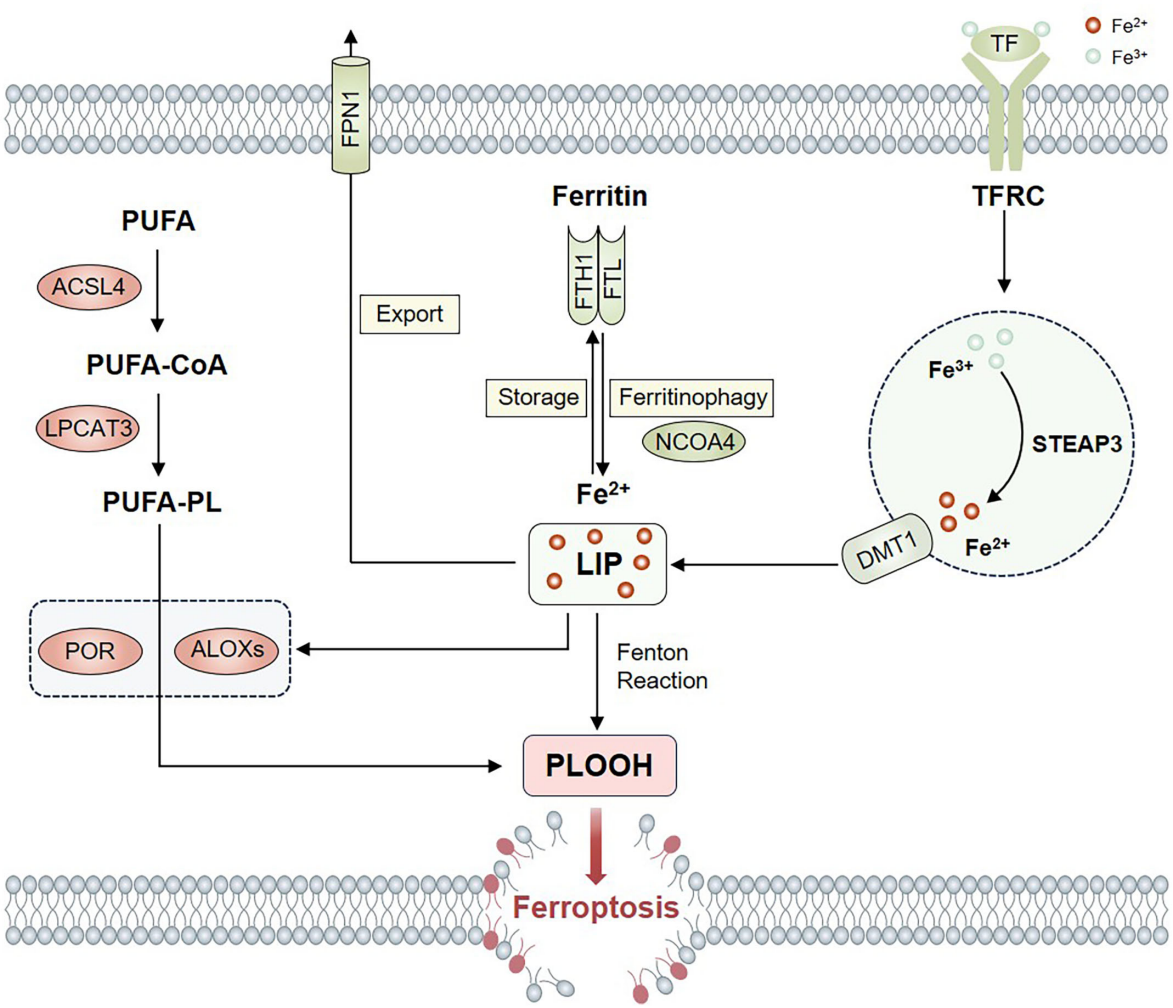
Lipid and iron metabolism in ferroptosis. ACSL4 and LPCAT3 promote the incorporation of PUFAs into phospholipids to form PUFA–PLs, which are vulnerable to oxidation mediated by ALOXs and POR. Several proteins, including TF, TFRC, STEAP3, DMT1, FPN1, FTH1, FTL and NCOA4 control ferroptosis through the regulation of iron metabolism. The iron released from LIP promote ROS accumulation through the Fenton reaction. ALOXs and POR also require iron for catalysis.

Lipid peroxidation is regarded as the key driving factor of ferroptosis (27) (**Figure 2**). Polyunsaturated fatty acids (PUFA), particularly adrenic acid and arachidonic acid, are prone to lipid peroxidation and play a crucial role in the execution of ferroptosis (28–30). Acyl-CoA synthetase long-chain family member 4 (ACSL4) and lysophosphatidylcholine acyltransferase 3 (LPCAT3) are associated with the biosynthesis and remodeling of PUFAs in cellular membranes and are important drivers of ferroptosis. ACSL4 catalyzes the linking of coenzyme A to long-chain PUFAs, and these products are esterified to phospholipids by LPCATs (31). Four LPCAT isoforms are known to date, among which LPCAT3 is able to preferentially catalyze the reversible exchange of fatty acids between the sn-2 position of lysophosphatidylcholine and the acyl-CoA pool, thereby increasing long-chain PUFAs to engage in the synthesis of membrane phospholipids (32). Thus, ACSL4 and LPCAT3 may regulate ferroptosis by regulating lipid metabolism. Studies have shown that inhibiting ACSL4 expression can prevent lipid peroxide accumulation and enhance cellular resistance to ferroptosis (29, 33). On the contrary, upregulating the expression of ACSL4 or increasing its activity can induce ferroptosis (34, 35). Similarly, supplementation with arachidonic acid or other PUFAs can increase cellular sensitivity to ferroptosis (17).

Lipoxygenases (ALOX) are non-heme iron-containing oxygenases that can directly oxidize PUFAs in cellular membranes to produce lipid peroxides, suggesting that ALOXs may mediate the occurrence of ferroptosis (36). Silencing ALOXs gene makes cells resistant to erastin-induced ferroptosis (28), and ALOXs inhibitors such as baicalein can effectively inhibit RSL3-induced ferroptosis (37). Research indicates that the universally expressed enzyme cytochrome P450 oxidoreductase (POR) is associated with the initiation of lipid peroxidation (38). Upon receiving electrons from POR, with NADPH serving as the electron donor, downstream electron acceptors such as cytochrome b5 type A and cytochrome P450 undergo reduction, which could subsequently induce lipid peroxidation through reducing ferric iron or removing methylene hydrogen from PUFAs (38, 39). The degree of ferroptosis can be evaluated by measuring the end products of lipid peroxidation, such as malondialdehyde and 4-hydroxynonenal (40).

The distribution and content of PUFAs influence the level of lipid peroxidation within the cell, thereby affecting the extent of ferroptosis. These findings represent an additional regulatory mechanism for ferroptosis, suggesting that prospective research could modulate ferroptosis by regulating the enzymes responsible for synthesizing membrane phospholipids containing PUFA.

### 2.3 Iron metabolism

Iron homeostasis is maintained by meticulously regulating the processes of iron metabolism, encompassing iron intake, storage, export, and utilization. Any imbalance in these processes may result in iron overload and subsequent ferroptosis (18) (**Figure 2**). In plasma, Fe^3+^ binds to transferrin (TF), and the iron-laden TF is subsequently endocytosed into the cell through the transferrin receptor (TFRC) located on the cell membrane, ultimately becoming localized within the endosome (41). The six-transmembrane epithelial antigen of the prostate 3 (STEAP3) catalyzes the reduction of Fe^3+^ to Fe^2+^ within the endosome. Subsequently, Fe^2+^ is translocated into the cytoplasm by the divalent metal transporter 1 (DMT1) (42, 43). Ferroportin 1 (FPN1) is the sole identified mammalian iron-exporting protein responsible for exporting excess Fe^2+^ from the cytoplasm to the outside (44). The majority of intracellular iron is stored as Fe^3+^ in ferritin, a protein complex that consists of ferritin light chain (FTL) and ferritin heavy chain 1 (FTH1). A minor proportion of iron is present as free iron ions within the cytoplasm, referred to as the labile iron pool (LIP). Iron released from the LIP promotes the accumulation of ROS via the Fenton reaction, ultimately leading to ferroptosis (45). In addition, the metabolic enzymes related to lipid peroxidation, ALOXs and POR, also need iron for catalysis (31).

Therefore, iron metabolism-related proteins are closely related to the regulation of ferroptosis. Research has demonstrated that increased expression of TFRC leads to iron overload and then induce ferroptosis (46). Silencing TFRC inhibits erastin-induced ferroptosis (22), whereas a reduction in FPN1 expression enhances cellular susceptibility to ferroptosis (47, 48). Depletion of ferritin results in the release of iron into the LIP, thereby increasing cellular susceptibility to ferroptosis. Conversely, more ferritin leads to increased stored iron and less iron in the LIP, thus enhancing cellular resistance against ferroptosis (49). Nuclear receptor coactivator 4 (NCOA4), a selective cargo receptor, delivers ferritin to lysosomes and releases ferritin-bound iron into LIP, which maintains iron homeostasis by regulating the autophagy of ferritin (50). Inhibiting the expression of NCOA4 may reduce the degradation of FTH1 and resist erastin-induced ferroptosis. Conversely, the overexpression of NCOA4 may reduce the levels of FTH1 and induce ferroptosis (51). In summary, regulation of iron metabolism and ferritinophagy are other potential targets for regulating ferroptosis.

## 3 Ferroptosis-related gene in thyroid cancer

Most ferroptosis-related genes exhibited differential expression between normal thyroid tissues and thyroid cancer tissues (52). Several researchers have investigated the prognostic significance of ferroptosis-related genes as potential biomarkers in patients with thyroid cancer. At present, dozens of differentially expressed genes have been identified as independent prognostic factors in patients with thyroid cancer (**Table 1**). We will discuss the roles of key ferroptosis modulators in thyroid cancer in the following sections.

**Table 1:** Ferroptosis-related genes as prognostic biomarkers for thyroid cancer.

Gene name	Data source	Diagnostic value	Prognostic indicator	Ref.
DPP4, GPX4, GSS, ISCU, MIOX, PGD, TF, and TFRC	TCGA database	The AUCs at 1, 2, and 3 years were 0.947, 0.907, and 0.886, respectively.	OS	(52)
AKR1C3	GEO database	The AUCs at 1, 2, and 5 years were 0.941, 0.945, and 0.795, respectively.	OS	(103)
HSPA5, AURKA, and TSC22D3	TCGA database	The AUCs at 1, 2, and 3 years were 0.780, 0.784, and 0.676, respectively.	PFS	(104)
DPP4, TYRO3, TIMP1, CDKN2A, SNCA, NR4A1, IL-6, and FABP4	TCGA database	The AUCs at 1, 3, and 5 years were 0.869, 0.755, and 0.844, respectively.	OS	(105)
DPP4, GSS, HMGCR, PGD, and TFRC	TCGA database	The AUCs at 1, 2, and 3 years were 0.621, 0.728, and 0.875, respectively.	OS	(54)
Ac008063.2, APOE, BCL3, ACAP3, ALOX5AP, ATXN2L, and B2M	TCGA database	The AUC of the ROC curve was 0.748.	/	(106)
ANGPTL7, CDKN2A, DPP4, DRD4, ISCU, PGD, SRXN1, TF, TFRC, and TXNRD1	TCGA database	The AUCs at 1, 3, and 5 years were 0.949, 0.900, and 0.859, respectively.	OS	(56)
AKR1C1, DPP4, GPX4, GSS, HMGCR, TFRC, SQLE, and PGD	TCGA database	The AUCs at 1, 2, and 3 years were 0.887, 0.890, and 0.842, respectively.	OS	(55)

### 3.1 Iron

Excess iron promotes the generation of ROS and induces ferroptosis. Meanwhile, iron is also essential trace element for thyroid hormone synthesis and metabolism (53). However, no studies have compared iron levels between thyroid cancer and normal thyroid tissues. Several recent studies have reported changes in the expression of iron metabolism-related proteins in thyroid cancer (52, 54–56). Iron in plasma is captured by TF and the iron-bearing TF is transported into the cell via TFRC. Research indicated that the expression of TFRC was increased in thyroid cancer tissues compared with normal tissues (54, 55). However, Yang et al. showed that TFRC was downregulated in PTC tissues (52). Shi et al. found that the expression of TFRC in PTC tissues and normal tissues was not significantly different (56). Therefore, the level of TFRC expression in thyroid cancer remains controversial. Could the differences in TFRC expression across various studies be related to tumor stage or subtypes of thyroid cancer? Currently, no studies have reported the effect of regulating the TFRC expression on thyroid cancer development. Consequently, additional research is required to investigate the function of TFRC in thyroid cancer. In addition to iron transport, storage mechanisms also modulate ferroptosis in thyroid cancer. NCOA4 releases ferritin-bound iron into LIP and increases the iron level by ferritinophagy. The research demonstrated that the overexpression of SIRT6 resulted in an upregulation of NCOA4 expression, an increase in intracellular Fe^2+^ levels, and suppression of the growth of ATC (57). We can infer that NCOA4 releases iron into the LIP and exhibits a negative correlation with the progression of thyroid cancer. The role of iron in thyroid cancer is not yet fully understood and requires further investigation in future studies.

### 3.2 GPX4

The GPX4 protein is capable of preventing the accumulation of toxic lipid peroxides, thereby suppressing ferroptosis. Studies showed that the expression of GPX4 was higher in thyroid cancer tissues than in normal tissues (11, 55). Furthermore, Zhang et al. suggested that the upregulated GPX4 expression in thyroid cancer tissues might be related to epigenetic regulation (58). In addition, another study found a correlation between GPX4 overexpression and thyroid cancer progression. Thus, GPX4 can be regarded as a risk factor for the overall survival (OS) of patients with thyroid cancer. Further investigation demonstrated that GPX4 knockdown triggered ferroptosis and inhibited the proliferation of thyroid cancer cells (11). These findings indicate that GPX4 may promote tumorigenesis by inhibiting ferroptosis in thyroid cancer. Consequently, the inhibition of GPX4 may be a promising therapeutic strategy for thyroid cancer.

### 3.3 GSH

GSH exhibits strong antioxidative function. Any changes in the synthesis of GSH may affect ferroptosis. Solute carrier family 7 member 11 (SLC7A11) is an essential component of system X_c_^−^. Suppressing SLC7A11 leads to a reduction in GSH levels, thereby inducing ferroptosis (9, 24). The level of SLC7A11 mRNA is dramatically higher in PTC tissues than in noncancerous tissues. Experiments demonstrated that the overexpression of SLC7A11 promoted the migration and invasion of PTC cells, whereas the knockdown of SLC7A11 had the opposite effect (59). Moreover, the research showed that the overexpression of fat mass and obesity-associated protein (FTO) suppressed PTC development by downregulating SLC7A11 expression via inducing ferroptosis (59). The other study found that E26 transformation-specific variant 4 (ETV4), a transcription factor, was highly expressed in PTC cells and tissues. The data suggested that knocking down ETV4 inhibited PTC growth *in vivo* by downregulating SLC7A11. Conversely, overexpressing ETV4 resulted in the upregulation of SLC7A11 expression, increased GSH levels and promoted the growth of PTC xenografts in mice (60). GSS is the second enzyme involved in the biosynthesis of GSH (17). Increased GSS expression further enhances GSH synthesis (61). Studies showed that thyroid cancer tissues exhibited higher GSS expression compared with normal tissues (54, 55). Similarly, breast cancer tissues exhibited higher GSH levels compared with normal tissues, which was associated with an increased risk of breast cancer (62). The synthesis of GSH plays an important role in ferroptosis. We can infer that over-activation of GSH synthesis may inhibit ferroptosis in thyroid cancer and be related to thyroid cancer progression.

### 3.4 DPP4

DPP4, also termed CD26, is a glycoprotein predominantly located on the plasma membrane and can cleave and degrade numerous biologically active peptides (63). Research has confirmed that DPP4 can limit GSH levels, elevate lipid ROS, and ultimately lead to ferroptosis (64–66). The research revealed that the tumor suppressor p53 limited ferroptosis by suppressing the activity of DPP4. DPP4-NOX binding is essential for lipid peroxidation in ferroptosis (67). Studies showed that PTC tissues exhibited higher DPP4 expression compared to noncancerous tissues. Increased DPP4 levels were associated with higher risks of metastasis and poorer survival in PTC (68, 69). However, the observation remains controversial because DPP4 has been shown to activate ferroptosis. Thus, the impact of DPP4 on thyroid cancer still requires additional investigation.

## 4 Ferroptosis−related noncoding RNA in thyroid cancer

Noncoding RNA is RNA in the transcriptome that is not translated into proteins. Long non-coding RNA (LncRNA) contains more than 200 nucleotides. Increasing evidence has revealed the importance of lncRNAs for promoting or suppressing tumors (70). Recent studies have revealed that dysregulation of specific lncRNAs is closely related to the ferroptosis of malignant tumors (71). In addition, some findings have shown that lncRNAs are involved in the development of thyroid cancer (72). lncRNAs exhibit markedly differential expression profiles in patients with thyroid cancer compared to normal controls. Therefore, their differential expression and high specificity make them potential diagnostic and prognostic biomarkers for thyroid cancer. Qin et al. explored the prognostic significance of ferroptosis-related lncRNAs (FRL) in thyroid cancer patients. The FRLs (LINC00900, LINC02454, AC012038.2, DPP4-DT, and AC055720.2) were used to group patients into low-risk and high-risk groups. The results showed that patients classified as high-risk exhibited a worse prognosis compared to those in the low-risk group. Further analysis indicated that low-risk thyroid cancer patients exhibited significant activation of immune-related pathways against cancer (73). Researchers found that eleven lncRNAs (LINC02861, DPP4-DT, LINC02345, AC034213.1, RNF213-AS1, AC108449.2, AL033397.2, AL133367.1, SMIM25, AC079848.1, and BX322562.1) play a pivotal role in the prognosis of thyroid cancer. Meanwhile, they found a strong association between tumor immune microenvironment and FRL prognostic model (74). It can be concluded that FRLs may have potential functions in modulating tumor immune microenvironment, thereby further influencing thyroid cancer progression. lncRNA CERS6-AS1 has been found as a tumor promoting gene in breast, pancreatic, and liver cancer (75–77). The study found that LncRNA CERS6-AS1 facilitated oncogenesis and restrained ferroptosis in PTC. Additionally, downregulation of CERS6-AS1 reduced cell viability and amplified ferroptosis by regulating the miR-497-5p/LASP1 axis in PTC (78). In sum, targeting lncRNAs to modulate ferroptosis is a novel idea for thyroid cancer therapy.

Circular RNAs (circRNAs), a class of non-coding RNA, play crucial roles in modulating multiple biological processes involved in tumor progression. Chen et al. demonstrated that circKIF4A expression was upregulated in PTC, while circKIF4A downregulation resulted in reduced growth and migration of PTC. Further, it was determined that circKIF4A can directly sponge miR-1231 and facilitate the progression of PTC by upregulating the expression of GPX4 (79). Another study indicated that circ_0067934 suppressed ferroptosis in human FTC and PTC cell lines through miR-545-3p/SLC7A11 signaling pathway. Additionally, the silencing of circ_0067934 inhibited thyroid cancer cell proliferation (80). The findings indicate that ferroptosis−related noncoding RNAs may serve as potential therapeutic targets by modulating ferroptosis, which provides novel insights into the treatment of thyroid cancer.

## 5 Ferroptosis in thyroid cancer therapy

**Figure 3:**
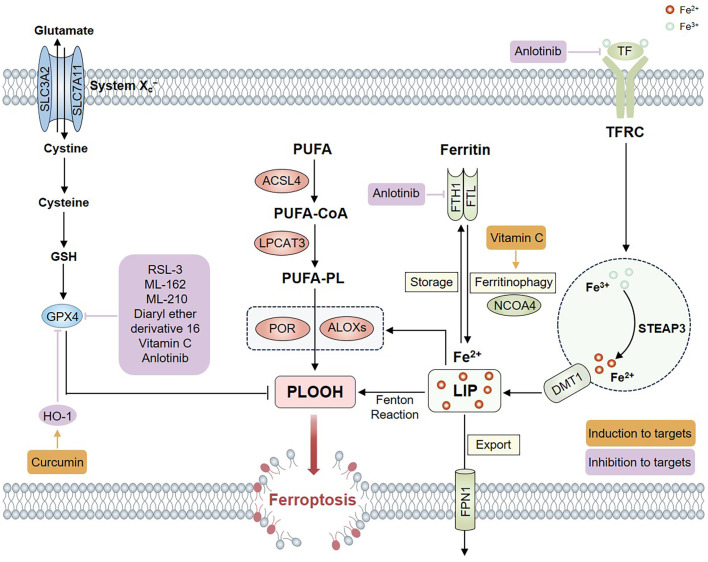
Targeting ferroptosis in thyroid cancer therapy. Regulated targets of ferroptosis-related agents in thyroid cancer therapy are shown. The positive regulators for targets are included in yellow boxes, and the negatives regulators are in purple boxes.

Lots of research has shown that ferroptosis-related genes and non-coding RNAs play pivotal roles in thyroid cancer progression. Some agents that induce ferroptosis have exhibited significant potential in thyroid cancer therapy (**Figure 3**).

Ferroptosis inducers have been proven to have therapeutic effects in various tumors. The study showed that RSL3, a direct inhibitor of GPX4, significantly activated ferroptosis, enhanced DNA damage, impaired DNA repair mechanisms, and suppressed thyroid cancer cell survival (81). In addition, researchers showed that GPX4 inhibitors (ML-162, ML-210, and RSL-3) induced ferroptosis and inhibited proliferation in RAS or BRAF-mutant PTC cells with TERT promoter and PIK3CA co-mutations (82). Pamarthy et al. found that diaryl ether derivative 16, a new small molecule, may trigger ferroptosis. The results showed that the diaryl ether derivative 16 reduced thyroid cancer cell proliferation and induced ferroptosis by suppressing GPX4 expression (83). Therefore, ferroptosis inducers may constitute an attractive strategy for thyroid cancer treatment. Further investigation is required to evaluate the efficacy and potential side effects of ferroptosis inducers in various subtypes of thyroid cancer.

Vitamin C, a scavenger of free radicals, can protect healthy cells from oxidative damage in humans (84). However, vitamin C at pharmacological concentrations is able to promote the production of hydroxyl radicals through the Fenton reaction (85). Therefore, it may induce ferroptosis in cancer cells. Increasing evidence has demonstrated that vitamin C has antineoplastic activity (86). The recent research indicated that vitamin C could effectively suppress the growth and long-term proliferation of ATC cells by activating ferroptosis. The main mechanisms were that vitamin C treatment induced GPX4 inactivation as well as the positive feedback, including ferritinophagy activation, iron accumulation, and ROS generation (87). In pancreatic cancer cells, pharmacological vitamin C could significantly enhance sensitivity to erastin-induced ferroptosis involving ferrous iron accumulation and GSH reduction, while inhibiting the cytotoxicity in normal cells (88). These studies suggest that vitamin C may play an anticancer role by inducing ferroptosis. The ability of vitamin C to induce ferroptosis in cancer cells is closely related to its concentration. More investigations are needed to explore the optimal concentration of vitamin C in thyroid cancer treatment.

Some herbal extracts can suppress the growth of tumors via regulating ferroptosis. Neferine, a predominant bisbenzylisoquinoline alkaloid derived from the seed embryos of lotus, exhibits various pharmacological properties, including antioxidant, anti-inflammatory, and anti-tumor effects (89). Research has demonstrated that neferine exhibits therapeutic effects on prostate cancer, lung cancer and cervical cancer (90–92). Li et al. found that neferine exhibited both ferroptosis-inducing and anti-tumor effects on human PTC and ATC cell lines via suppressing the Nrf2/HO-1/NQO1 signaling pathway (93). Curcumin extracted from the roots of Curcuma longa has exhibited anticancer, anti-inflammatory, antioxidant, and hypoglycemic effects (94). Recent research has demonstrated that curcumin can suppress breast cancer cells and non-small cell lung cancer by inducing ferroptosis (95, 96). The recent study showed that curcumin suppressed the growth of FTC through HO-1-induced activation of ferroptosis. Further studies indicated that HO-1 induced ferroptosis by inhibiting GPX4 expression (97). Similarly, treatment with curcumin resulted in a significant upregulation of HO-1 and a downregulation of GPX4 in breast cancer cells. According to the Comparative Toxicogenomics Database, curcumin may directly target the HO-1 gene (95). HO-1 plays a bifunctional role in the regulation of ferroptosis. Generally, Nrf2-derived HO-1 inhibits ferroptosis and exerts a cytoprotective effect by neutralizing ROS when HO-1 is activated moderately. However, HO-1 overactivation induces ferroptosis due to the excessive accumulation of labile Fe^2+^ (98). Neferine and curcumin possess various pharmacological activities. Additional research is required to investigate the translational potential of neferine and curcumin in targeting ferroptosis, thereby providing more clinical evidence for thyroid cancer treatment.

Recent studies have demonstrated that certain traditional drugs used in thyroid cancer treatment can induce ferroptosis. Anlotinib is an antiangiogenic multikinase inhibitor that targets fibroblast growth factor receptor 1, platelet-derived growth factor receptor, and vascular endothelial growth factor receptor 2 (99). A Phase 1 clinical trial demonstrated that anlotinib exhibited antitumor efficacy against medullary thyroid cancer and non-small cell lung cancer (100). The previous study showed that anlotinib suppressed the viability, proliferation, and migration of ATC cells. Further, anlotinib significantly reduced the expression of ferroptosis-related genes, including GPX4, FTL, FTH1, HO-1, and TF. In addition, anlotinib activated protective autophagy, while the blockade of autophagy enhanced anlotinib-mediated ferroptosis and antitumor effects (101). The study indicated that anlotinib may treat ATC by inducing ferroptosis, and the autophagy-ferroptosis signaling pathway may offer a synergistic combination treatment strategy. Sorafenib is an FDA-approved medication used in thyroid cancer treatment. The precise mechanisms through which sorafenib induces ferroptosis have been thoroughly studied only in hepatocellular carcinoma (102). Additional studies are required to explore whether sorafenib induces ferroptosis in thyroid cancer. In summary, triggering ferroptosis represents a viable and effective antitumor strategy, providing novel insights into thyroid cancer therapy.

## 6 Conclusion and perspectives

This review outlines the core mechanisms of ferroptosis and discusses the roles of ferroptosis-related modulators, including iron, GPX4, GSH, and DPP4, in thyroid cancer. Furthermore, we explored the association between ferroptosis and therapeutic agents used for thyroid cancer. Although ferroptosis is considered an important target for thyroid cancer therapy, there are still many challenges to its clinical application.

Firstly, most of the current research focuses on the prognostic value of ferroptosis-related genes as biomarkers in patients with thyroid cancer. Limited research has been conducted on ferroptosis in thyroid cancer to elucidate the comprehensive control mechanisms. The exact relationship between ferroptosis and thyroid cancer needs to be further studied. Second, the value of utilizing ferroptosis inducers alongside conventional anti-thyroid cancer drugs remains uncertain. Given the involvement of various cell death modalities, such as necroptosis, autophagy, and apoptosis, in thyroid cancer treatment, it would be highly beneficial to explore the effects of combination therapies that target distinct cell death pathways. Finally, ferroptosis is also observed in normal tissues. Consequently, inducing ferroptosis in thyroid cancer treatment may lead to certain complications. It is important to study new drug delivery systems for targeted delivery of ferroptosis inducers to tumor cells. Additionally, further research should be conducted to identify more ferroptosis-related regulators, with the aim of discovering markers that can effectively select suitable patients.

In conclusion, ferroptosis indisputably plays a significant role in thyroid cancer therapy. The comprehensive molecular mechanisms and the underlying signaling pathways of ferroptosis in thyroid cancer need further investigation. These studies may offer new insights into thyroid cancer therapy. To date, the therapeutic effects of ferroptosis inducers in thyroid cancer have primarily been demonstrated in animal experiments, and their clinical translation remains challenging. Therefore, well-designed clinical studies are essential to evaluate the potential of ferroptosis-based therapies in thyroid cancer. Moreover, the combination of conventional anti-tumor strategies with ferroptosis-inducing agents holds promise for enhancing the efficacy of thyroid cancer treatment.
